# An automatic system for the simultaneous translation of lectures

**DOI:** 10.1186/1758-2946-6-S1-O7

**Published:** 2014-03-11

**Authors:** Sebastian Stüker

**Affiliations:** 1 Institut für Anthropomatik (IFA), Adenauerring 2, 76135 Karlsruhe, Germany

## 

Lectures at Karlsruhe Institute of Technology (KIT) are mainly taught in German. Therefore, foreign students that want to study at KIT need to learn German, and not only at a conversational level, but must be proficient enough to follow highly scientific and technical lectures carrying complex content. While foreign students often take a one year preparatory course that teaches them German, experience shows that even after that course, their German is not proficient enough to be able to follow German lectures and thus perform well.

Since the use of human interpreters for bridging the language barrier in lectures is too expensive, we want to solve this issue with the help of our automatic simultaneous lecture translation system. In this system we employ the technology of spoken language translation (SLT), which combines automatic speech recognition (ASR) and machine translation (MT) to build a system that simultaneously translates lectures from German to English.

In this, university lectures are characterized by a multitude of diverse topics and a large amount of technical terms. This poses specific challenges to a speech translation system, e.g., a very specific vocabulary and language model are needed. Also, in order to be able to translate simultaneously, i.e., to interpret the lectures, the components of the systems need special modifications.

Our system works with the help of a cloud based service infrastructure. The speech of the lecturer is recorded via a lo cal client and sent to the service infrastructure. A service then manages the flow of the data through the ASR, MT, and other components. The output of the system is delivered in the form or real- time subtitles via a web site that can be accessed by the students attending the lecture through mobile phones, tablet computers or laptops.

